# Two-Step Sintering of Partially Stabilized Zirconia for Applications in Ceramic Crowns

**DOI:** 10.3390/ma13081857

**Published:** 2020-04-15

**Authors:** Bobby Aditya Darmawan, John G. Fisher, Doan Thanh Trung, Kumaresan Sakthiabirami, Sang-Won Park

**Affiliations:** 1 School of Materials Science and Engineering, Chonnam National University, Gwangju 61186, Korea; 2 Department of Prosthodontics, Dental Science Research Institute, School of Dentistry, Chonnam National University, Gwangju 61186, Korea

**Keywords:** zirconia, two-step sintering, microstructure, translucency

## Abstract

Partially-stabilized zirconia is used in ceramic crowns due to its excellent mechanical properties and bio-inertness but does not match the natural color and translucency of tooth enamel. To reduce scattering of light and improve translucency, the grain size of zirconia ceramics should be less than the wavelength of visible light (0.4–0.7 μm), and porosity should be eliminated. The aim of the present work was to study the effect of two-step sintering of a commercial powder (Zpex Smile, Tosoh Corp., Tokyo, Japan) on the grain size and translucency of zirconia for use in ceramic crowns. Samples were sintered at a first step temperature (T_1_) of 1300, 1375 and 1400 °C for 5 min, followed by a decrease to the second step temperature (T_2_) and holding at T_2_ for 5–20 h. Samples were also conventionally sintered at 1450 °C for 2 h for comparison. Two-step sintered samples with an almost equal density, smaller grain size and narrower grain size distribution compared to conventionally sintered samples could be sintered. However, the translucency of two-step sintered samples had lower values compared to conventionally sintered samples. This is due to the slightly higher porosity in the two-step sintered samples. Density and translucency of both conventionally and two-step sintered samples could be increased further by using a ball milled powder.

## 1. Introduction

Yttria-stabilized tetragonal zirconia polycrystalline (Y-TZP) ceramics are used as ceramic crowns due to their advantageous properties, such as biocompatibility, esthetics, strength, durability and ease of customization [1,2]. However the optical properties of zirconia do not match those of tooth enamel, limiting their application. In particular, Y-TZP ceramics are opaque, whereas tooth enamel is translucent [3,4]. The esthetic properties of Y-TZP ceramics can be improved using veneering with porcelain, but this can cause a reduction in mechanical properties, due to fracture of the porcelain veneer [2,5,6,7]. This has led to research to improve the translucency of Y-TZP ceramics to allow their use as monolithic restorations without the need for porcelain veneer [8,9].

The translucency of Y-TZP ceramics is controlled by absorption and scattering of light as it passes through the ceramic body. When light encounters any interface between media where there is a change in refractive index, reflection and refraction take place, leading to scattering. Light is scattered by porosity, by secondary phases and at grain boundaries if the material is optically anisotropic, i.e., has a non-cubic unit cell [10,11,12,13,14,15,16]. To prepare ceramics with high translucency, it is necessary to sinter to high density (>99.9% theoretical density), avoid the presence of secondary phases (particularly at the grain boundaries) and, in the case of an optically anisotropic material such as Y-TZP, to keep the grain size small with respect to the wavelength of visible light [12,13,15,16,17]. Porosity is considered to be the main cause of scattering [10,11,13], but as pore size is related to grain size [11,18], a reduction in grain size is still expected to be helpful in reducing scattering. The translucency of Y-TZP ceramics can also be improved by lowering the amount of alumina additive (added to increase resistance to low temperature degradation [19,20,21]) and by preparing tetragonal/cubic zirconia composite materials [17,22,23,24,25]. However, reducing alumina content and increasing the amount of cubic zirconia phase will reduce the low temperature degradation resistance and toughness of zirconia [21,24,26]. Hence, optimizing sintering parameters in order to obtain samples with high density and fine grain size is a topic of interest.

The fabrication of Y-TZP ceramics is usually carried out using solid-state sintering at a high temperature (1350–1550 °C) [26,27,28]. The high sintering temperature promotes not only densification but also grain growth [29,30,31] and the formation of the cubic phase of zirconia [26,27]. Since the properties of polycrystalline ceramics are controlled by the microstructure, it is important to control the grain growth while maintaining high density. However, highly dense ceramics with micro- or nanometer grain size are difficult to achieve with conventional sintering [30].

Processes such as reducing the concentration of sintering additives such as alumina [22], pressure-assisted sintering [32,33,34,35] and spark plasma sintering [36,37,38,39,40] could be alternative ways to prepare dense Y-TZP ceramics with fine grain size. Changing the concentration of sintering additives may have effects on other properties such as aging resistance [21,41], and pressure-assisted sintering and spark plasma sintering may not be cost-effective since the processes are more complex, expensive, and difficult to apply [30]. In addition, pressure-assisted sintering and spark plasma sintering are not suitable for sintering objects of complex shape. Hence, alternative methods of sintering Y-TZP to high density whilst maintaining a fine grain size have been sought.

Another sintering method to control the grain growth during densification is called two-step sintering. Two-step sintering was introduced in the early 1990s by Chu et al. [42]. Their technique used a low temperature pretreatment stage followed by a high temperature sintering stage. Later, a modification of two-step sintering was suggested by Chen and Wang, and this method has been more widely used [30,43]. According to their method, the first step temperature (T1) is at a relatively high temperature with a dwell time close to zero, followed by rapid cooling to the second step temperature (T2). In the first step, it is necessary to expose the sample to a temperature high enough to activate densification, with a density of at least 75% theoretical density (TD) required after the end of the first step. In the second step, the material is held at low temperature (T2) and exposed to a prolonged time defined as t2. This condition induces the further densification with minimal grain growth by exploiting the difference between grain boundary diffusion kinetics and grain boundary migration kinetics [43]. The two step sintering method of Chen and Wang has been successfully used to sinter many types of ceramic to high density while retaining a finer grain size than is possible using conventional sintering [30,44,45,46,47,48,49,50,51].

The two-step method has also been used to sinter Y-TZP [50,52,53,54,55,56]. The effects of two-step sintering on microstructure, density and ageing resistance have been investigated, but as far as we know, the effect of two-step sintering on the translucency of Y-TZP has not yet been studied. The aim of this study was to analyze the effect of conventional and two-step sintering schedules on the density, microstructure, translucency and mechanical properties of a commercial Y-TZP powder. Our hypothesis in this study was that a two-step sintering process would decrease the grain size while maintaining high density and lead to an increase in the translucency.

## 2. Materials and Methods

A commercial ZrO_2_-9.3 wt % (5.3 mol%) Y_2_O_3_ powder (Zpex Smile, Tosoh, Tokyo, Japan) was used in this study. The Al_2_O_3_ content of the powder is 0.05 wt %, SiO_2_ content is ≤0.02 wt % and Fe_2_O_3_ content is ≤0.01 wt %. Powder was uniaxially pressed with a pressure of 17.3 MPa into disks with a thickness of 2.1 mm and diameter of 19 mm, followed by cold isostatic pressing with a pressure of 147 MPa for 10 min. To find the first step temperature, T1, preliminary studies were conducted. Samples were sintered at temperatures in the range of 1000–1400 °C for 5 min with heating and cooling rates of 5 °C·min^−1^. Sample density was measured using the Archimedes method using deionized water. For microstructural examination, samples were sectioned vertically using a low speed diamond wheel saw and then polished to a 1 μm finish. Samples were thermally etched at a temperature 50 °C lower than the sintering temperature and the microstructure examined in a scanning electron microscope (SEM, S-4700, Hitachi High-Technologies, Tokyo, Japan). Mean grain size and grain size distribution were calculated from the SEM images using ImageJ software (National Institute of Mental Health, Bethesda, MD, USA). For each sample, at least 300 grains were measured and the results plotted in terms of equivalent 2D spherical diameter.

Based on the preliminary studies, the details of the first step temperature (T1), the second step temperature (T2) and the holding times at both steps (t1 and t2) for the two-step sintering schedules are summarized in Table 1. All heating and cooling rates were 5 °C·min^−1^. Samples conventionally sintered at 1450 °C for 2 h with heating and cooling rates of 5 °C·min^−1^ were used as a control group. Sample density and microstructure were examined as before. For the two-step sintered samples, a thermal etching temperature 50 °C lower than T2 was used.

X-ray diffraction (XRD) patterns of selected specimens were measured using a high-resolution X-ray diffractometer (XRD, X’Pert PRO, PANalytical, Almelo, the Netherlands) with Cu target and 2 kW power. MDI Jade 6 software (Materials Data Inc., Livermore, CA, USA) was used to analyze the XRD peaks based on ICDD cards #86-1450 and #80-0784 for monoclinic zirconia and tetragonal zirconia, respectively. The Vickers hardness of selected specimens was measured using a microhardness tester (Nova 130, Innovatest, Maastricht, the Netherlands) with five indentations for each sample. Samples were polished on one face using diamond disks of #600, #800, #1200 and #2400 grade. An indentation load of 1 kgf (9.807 N) was applied for 10 s on the polished faces. For each group measured, five samples were tested. The biaxial flexural strength of selected samples was measured at room temperature using a universal testing machine (RB model 301 Unitech MTM, UM-K100, R&B Inc., Daejeon, Korea) according to ISO standard 6872. Specimens were supported on three symmetrically placed hardened steel balls (3 mm in diameter) which were put in a support circle (12 mm in diameter). The load at a rate of 1 mm·min^−1^ was applied to the center of the top surface of the specimens by a piston (1 mm in diameter) until fracture occurred. The load to failure (N) of each specimen was recorded, and the biaxial flexural strength (MPa) was calculated according to ISO standard 6872 using the following equations [57]:

*S* = −0.2387 *P* (*X* − *Y*)/*d*^2^ (1)

where *S* is the maximum tensile stress (MPa), *P* is the load to failure (N) and *d* is the specimen thickness at the fracture origin (mm). *X* and *Y* were determined using the following formulae:

*X* = (1 + *ν*) *ln* (*r_2_/r_3_*)^2^ + [(1 − *ν*)/2] (*r_2_/r_3_*)^2^ (2)

*Y* = (1 + *ν*) [1 + *ln* (*r_1_/r_3_*)^2^] + (1 − *ν*) (*r_1_/r_3_*)^2^ (3)

where *ν* is Poisson’s ratio (a value of 0.25 was used as Poisson’s ratio of zirconia is not known), *r_1_* is the radius (in mm) of the support circle, *r_2_* is the radius (in mm) of the loaded area and *r_3_* is the radius (in mm) of the specimen. For each group measured, eight samples were tested. Specimen thickness was 1.4–1.5 mm. Hardness and flexural strength were analyzed for statistical significance using a one-way analysis of variance (ANOVA), and Tukey’s method was applied for post-hoc comparison (α = 0.05). The analysis was performed using SPSS software (IBM SPSS Statistics version 23 for Windows, IBM, Armonk, NY, USA).

A spectrophotometer was used to measure the contrast ratio, which is indicative of translucency, of selected sample groups. Samples for translucency measurements were polished with #600 and #1200 grade diamond disks, followed by polishing with 1 µm UP film (R&B Inc., Daejeon, Korea). Polishing was performed on both faces of the samples and the final sample thickness was between 0.5 and 0.6 mm. The measurements of translucency were made using a spectrophotometer (CM 2600d, Konica Minolta, Tokyo, Japan) connected to a computer running color measurement software (SpectraMagic™ NX version 1.9, Konica Minolta, Japan). Three samples were prepared for each group to obtain the contrast ratios of the materials over a white background (L*w) and a black background (L*b), with 5 points measured for each sample. A translucency evaluation was performed with the CIE Lab L*a*b* color system. The calculation of translucency was based on the translucency parameter, TP, as per the following equation:

(4) $$TP={\left[{\left(L_{b}^{*}-L_{w}^{*}\right)}^{2}+{\left(a_{b}^{*}-a_{w}^{*}\right)}^{2}+{\left(b_{b}^{*}-b_{w}^{*}\right)}^{2}\right]}^{\frac{1}{2}}$$

where L* is lightness, a* is the red/green component, b* is the yellow/blue component and subscripts *w* and *b* indicate measurement against a white and black background respectively. Results were analyzed for statistical significance as before.

In order to further improve the density and translucency of the two-step sintered samples, experiments were carried out using powder which had been ball milled to break up the secondary particles. Thirty grams of Zpex Smile powder was weighed and then ball milled in high purity ethanol (99.9%) with zirconia media in a polypropylene jar for 24 h. After evaporating the ethanol with a hot plate and stirrer, the powder was ground in an agate mortar and pestle and sieved to pass through a 180 µm mesh. Samples were then prepared and sintered as before.

## 3. Results

**Figure 1:**
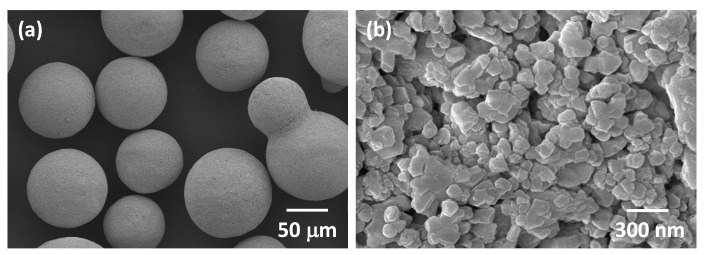
SEM micrographs of (**a**) secondary and (**b**) primary particles of as-received Tosoh Zpex Smile powder.

**Figure 2:**
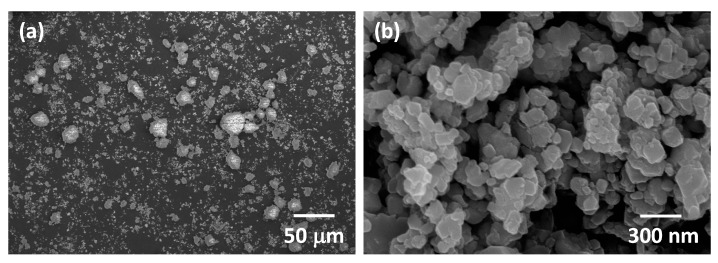
SEM micrographs of (**a**) secondary and (**b**) primary particles of Tosoh Zpex Smile powder after ball milling.

SEM micrographs of the as-received Tosoh Zpex Smile powder are shown in Figure 1. The powder consists of secondary particles up to 100 μm in diameter (Figure 1a). Each secondary particle consists of primary particles up to 300 nm in diameter (Figure 1b). SEM micrographs of the powder after ball milling are shown in Figure 2. The secondary particles have been partially broken up while the size of the primary particles remains unchanged (the white streaks in Figure 2a are caused by sample charging).

**Figure 3:**
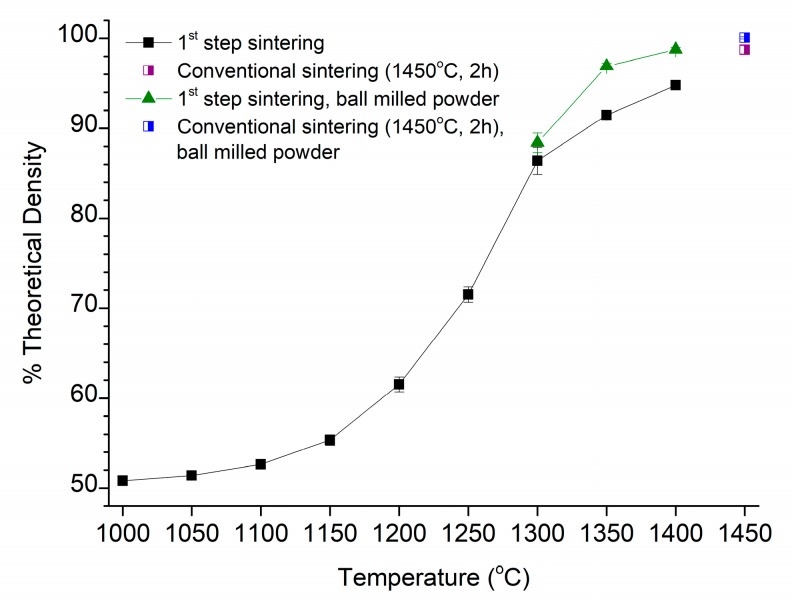
Density of samples after first step sintering vs. first step sintering temperature T1.

The density of the samples after the first step of sintering is shown in Figure 3 as a function of the first step temperature (T1). The theoretical density (TD) of the material was calculated to be 6.07 g·cm^−3^, based on the manufacturer’s composition and the unit cell volume calculated from an XRD pattern of a sample sintered at 1450 °C for 2 h. Sample density increases as T1 increases and samples sintered at T1 > 1250 °C have density values above 75% TD. After sintering at 1400 °C for 5 min, the sample density is already 94.8% TD. The density of samples conventionally sintered at 1450 °C for 2 h (98.7% TD) is shown for comparison. For the ball milled powder, first step sintering experiments were carried out at T1 = 1300, 1350 and 1400 °C (t1 = 5 min). Samples were also conventionally sintered at 1450 °C for 2 h. The density values of these samples are also shown in Figure 3. Ball milling the powder results in an increase in density, especially for the samples sintered at T1 = 1350 and 1400 °C. After ball milling, the density of the samples sintered at 1400 °C for 5 min is equal to that of the conventionally sintered samples prepared using as-received powder. The conventionally sintered samples also show increased density when ball milled powder is used; their density reaches 100% TD.

**Figure 4:**
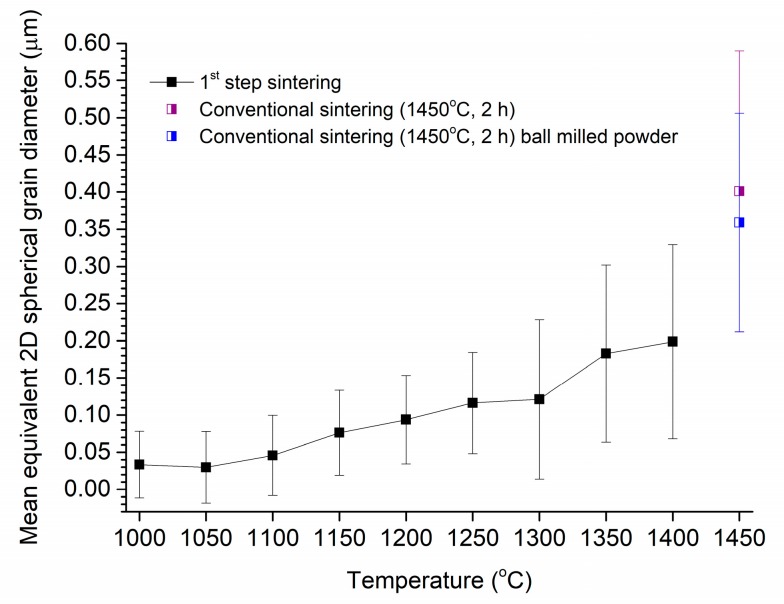
Mean grain size of samples after first step sintering vs. first step sintering temperature T1.

The mean grain size (diameter) of the samples after the first step of sintering is shown in Figure 4 as a function of the first step temperature, T1. Mean grain size increases gradually from a value of ~30 nm at 1000 °C to ~200 nm at 1400 °C. The mean grain sizes of samples conventionally sintered at 1450 °C for 2 h are also shown for comparison. The grain size of the samples after first step sintering is considerably lower than that of the conventionally sintered samples. The mean grain size of the conventionally sintered sample prepared from ball milled powder is slightly lower than that of the sample prepared from the as-received powder.

**Figure 5:**
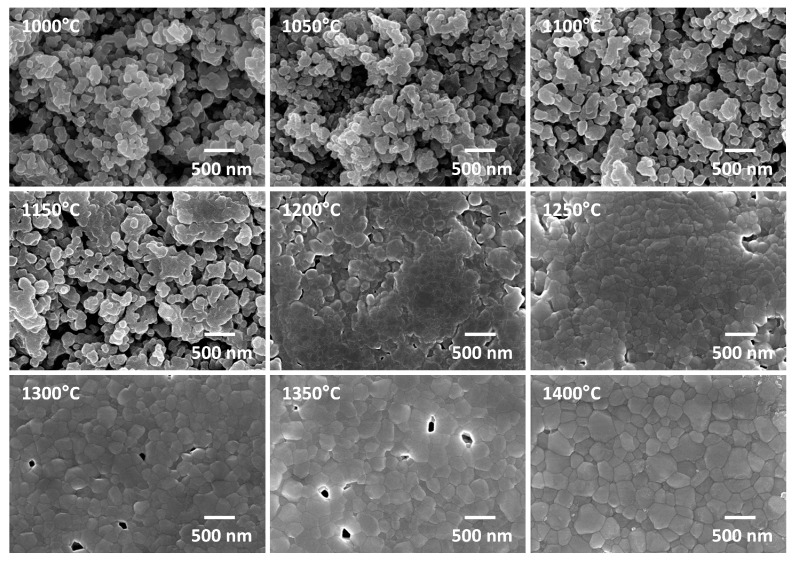
SEM micrographs of samples prepared from as-received powder after first step sintering. The temperatures in each label are T1.

Secondary electron imaging SEM micrographs of the samples prepared from as-received powder after the first step of sintering are shown in Figure 5. The temperatures in each label are T1. Samples sintered at temperatures from 1000 to 1100 °C are in the initial stage of sintering [58]. Necks have formed between the powder particles, but formation of grain boundaries is limited and the grain size barely changes with sintering temperature. Samples sintered from 1150 to 1250 °C are in the intermediate stage of sintering. Grain boundary networks have formed between the particles but extensive interconnected porosity still exists. As the sintering temperature increases, the degree of interconnected porosity steadily decreases and grain size slowly increases. Samples sintered at temperatures from 1300 to 1400 °C are in the final stage of sintering, with isolated pores between the grains. Grain size increases more rapidly, as there are now no pore channels to prevent grain growth.

**Figure 6:**
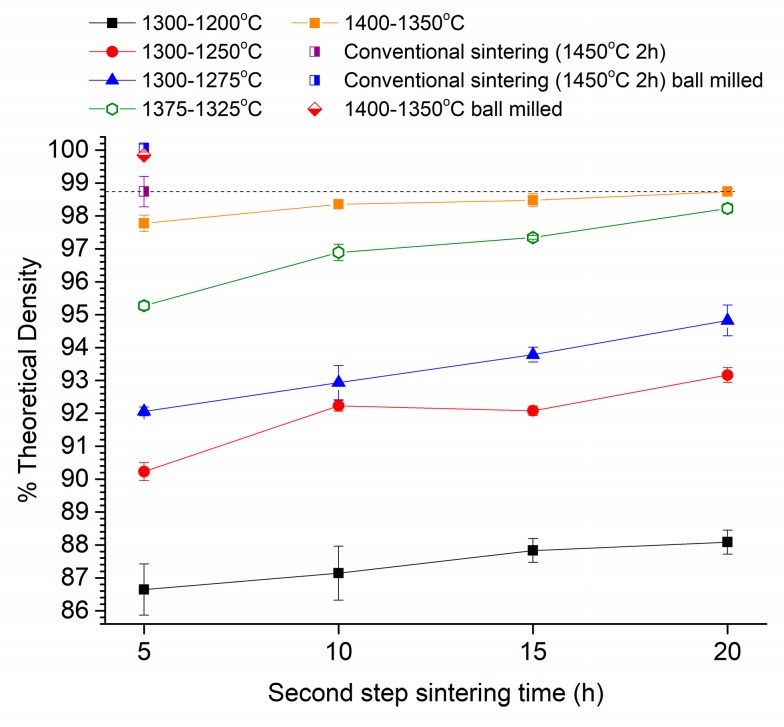
Density of samples after two-step sintering vs. second step sintering time t2.

The density of samples vs. second step sintering time (t2) of samples sintered using two-step sintering of the as-received powder are shown in Figure 6. The sample notation is in the form T1-T2, e.g., 1300-1200 °C means a first step temperature T1 = 1300 °C and a second step temperature T2 = 1200 °C. The density of samples conventionally sintered at 1450 °C for 2 h is shown for comparison. According to the density values in Figure 3, 1300 °C was chosen as the first step temperature due to the samples having a density of ~86% TD, higher than the value of 75% TD recommended by Chen and Wang. This was followed by choosing temperatures in the range of 1200–1275 °C as the second step temperature and sintering for 5–20 h (Table 1, schedule 1). The samples sintered with T2 = 1200 °C barely showed any densification, even after sintering for 20 h. Increasing the value of T2 caused improved densification, but even after increasing T2 to 1275 °C and t2 to 20 h, the density only reached 94.8% TD. This is much lower than the value of 98.7% TD for the conventionally sintered samples.

In order to increase sample density further, higher values of T1 were chosen (Table 1, schedules 2 and 3). Values 50 °C lower than T1 were then chosen for T2. An increase in T1 to 1375 °C allowed the density to reach 95.3% TD after sintering at T2 = 1325 °C for 5 h. Increasing t2 to 20 h allowed the density to reach 98.2% TD, slightly lower than that of the conventionally sintered samples. An increase in T1 to 1400 °C produced a density of 97.7% TD after sintering at T2 = 1350 °C for 5 h. However, further densification was sluggish and t2 had to be increased to 20 h for the sample density to reach the same value as that of the conventionally sintered samples, as shown by the dashed black line. The size of the error bars for the samples with T1 = 1400 °C and T2 = 1350 °C is smaller than those of the conventionally sintered samples, indicating that these two-step sintered samples show a lower variation in density amongst samples than those sintered under other schedules. Samples prepared from ball milled powder were sintered using the following two-step sintering schedule: T1 = 1400 °C; t1 = 5 min; T2 = 1350 °C; t2 = 5 h. Sample density was increased to 99.8% TD, almost equal to that of the conventionally sintered samples prepared from ball milled powder (Figure 6).

**Figure 7:**
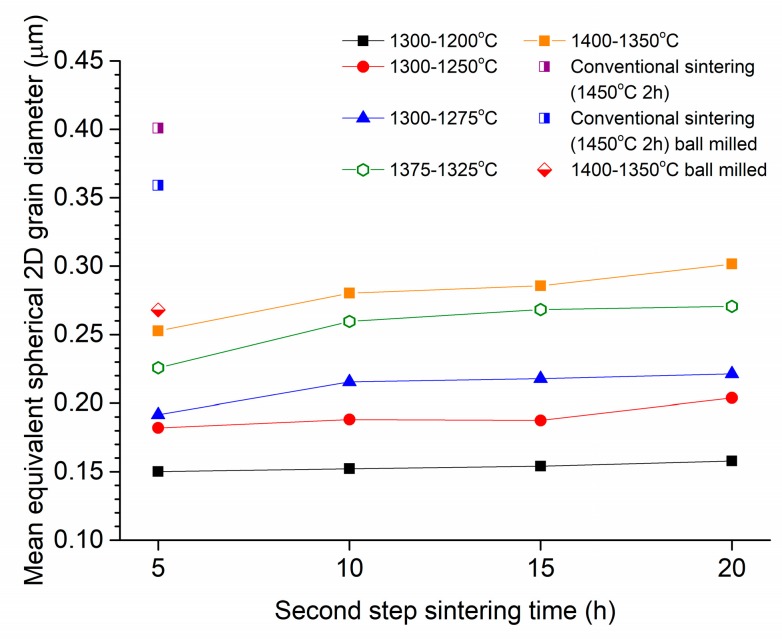
Mean grain size of samples after two-step sintering vs. second step sintering time.

The mean grain size of the samples after two-step sintering is shown in Figure 7. Error bars are not shown for clarity. For the samples prepared from as-received powder sintered under schedule 1 (T1 = 1300 °C, T2 = 1200–1275 °C), the mean grain size increases slightly as T2 increases. Mean grain size does not change much with second stage sintering time, t2, for the samples with T2 = 1200–1250 °C, but increases slightly for the samples with T2 = 1275 °C. Increasing T1 to 1375 and 1400 °C causes the mean grain size to increase further, and the mean grain size slowly increases with increased t2 time. In all cases, the mean grain size is lower than that of the conventionally sintered samples. The grain size of the two-step sintered sample prepared from ball milled powder is lower than that of the conventionally sintered samples and is similar to that of the corresponding two-step sintered sample prepared from the as-received powder (Figure 7).

**Figure 8:**
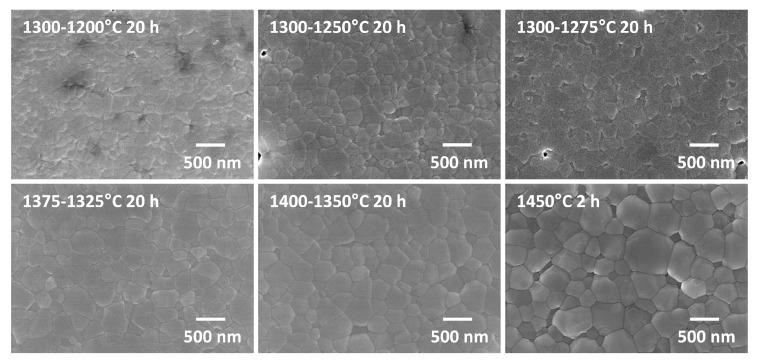
SEM micrographs of two-step sintered samples prepared from as-received powder. The temperatures in each label are T1 and T2 respectively.

**Figure 9:**
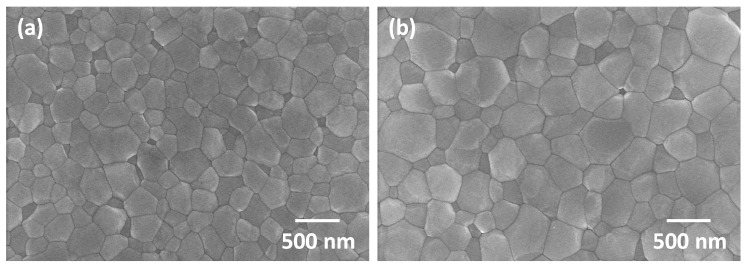
SEM micrographs of (**a**) a two-step sintered sample (T1 = 1400 °C, T2 = 1350 °C, t2 = 5 h) and (**b**) a conventionally sintered sample, both prepared using ball milled powder.

SEM micrographs of the two-step sintered samples prepared from as-received powder with t2 = 20 h are shown in Figure 8. The temperatures in each label are T1 and T2 respectively. A micrograph of a sample prepared from as-received powder and conventionally sintered at 1450 °C for 2 h is also shown. For the samples with T1 = 1300 °C, it can be seen that changing the value of T2 does not have much effect on grain size. Increasing the value of T1 to 1375 or 1400 °C has a more pronounced effect. It can also be seen that the conventionally sintered sample has larger grain sizes than the two-step sintered samples. SEM micrographs of the two-step sintered and conventionally sintered samples prepared from ball milled powder are shown in Figure 9. Again, the conventionally sintered sample has larger grain sizes than the two-step sintered sample.

**Figure 10:**
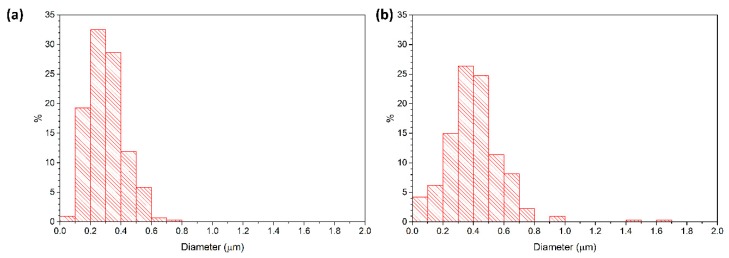
Grain size distributions of (**a**) a two-step sintered sample (T1 = 1400 °C, T2 = 1350 °C, t2 = 20 h) and (**b**) a conventionally sintered sample. Both samples prepared from as-received powder.

**Figure 11:**
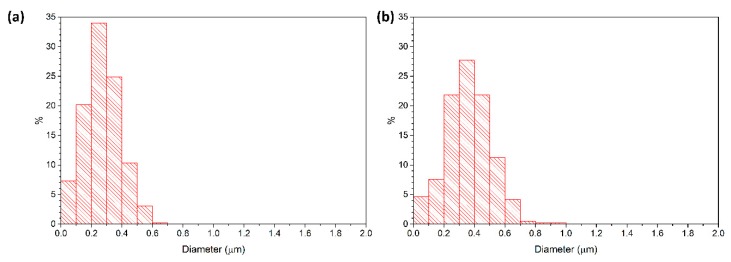
Grain size distributions of (**a**) a two-step sintered sample (T1 = 1400 °C, T2 = 1350 °C, t2 = 5 h) and (**b**) a conventionally sintered sample. Both samples prepared from ball milled powder.

Figure 10 shows the grain size distributions of a two-step sintered sample (T1 = 1400 °C, T2 = 1350 °C, t2 = 20 h) and a conventionally sintered sample, both prepared from as-received powder. Both samples have a density of 98.7% TD. The two-step sintered sample has a narrower grain size distribution than the conventionally sintered sample. The two-step sintered sample has a unimodal grain size distribution, whereas the grain size distribution of the conventionally sintered sample is slightly bimodal, with some grains 3–4 times larger than the mean grain size of 0.4 μm. Figure 11 shows the grain size distributions of a two-step sintered sample (T1 = 1400 °C, T2 = 1350 °C, t2 = 5 h) and a conventionally sintered sample, both prepared from ball milled powder. Both samples have slightly narrower grain size distributions than the corresponding samples prepared from the as-received powder. In addition, the grain size distribution of the conventionally sintered sample prepared from the ball milled powder is now unimodal rather than bimodal.

**Figure 12:**
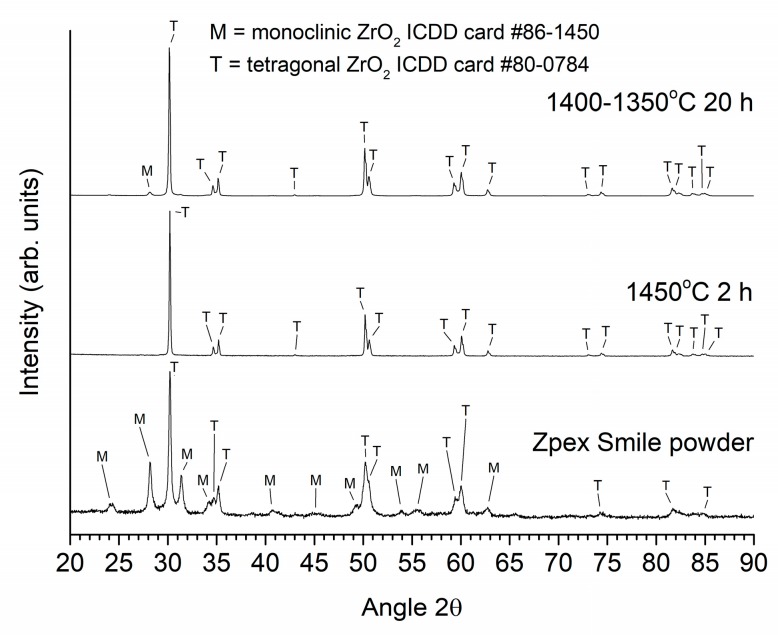
XRD patterns of the Zpex Smile powder, a conventionally sintered sample and a two-step sintered sample (T1 = 1400 °C, T2 = 1350 °C, t2 = 20 h). All samples prepared from as-received powder.

**Figure 13:**
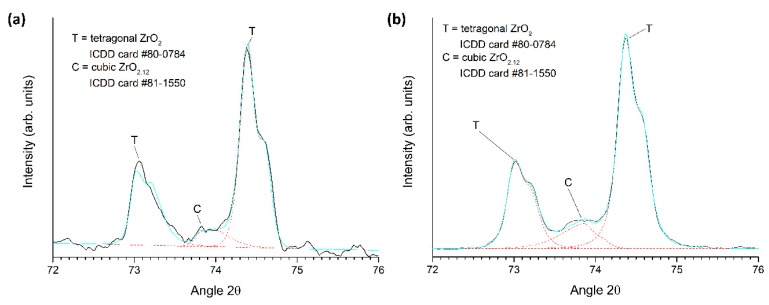
Magnified XRD patterns of (**a**) a conventionally sintered sample and (**b**) a two-step sintered sample (T1 = 1400 °C, T2 = 1350 °C, t2 = 20 h). Both samples prepared from as-received powder.

XRD patterns of the Zpex Smile powder, a conventionally sintered sample and a two-step sintered sample (T1 = 1400 °C, T2 = 1350 °C, t2 = 20 h), all prepared from as-received powder, are shown in Figure 12. Magnified views of the conventionally sintered and two-step sintered samples in the region 2θ = 72–76° are shown in Figure 13. Peak fitting in Figure 13 with Pearson-VII peaks was carried out using MDI Jade 6. The black lines are the original data, the red dashed lines are the background and fitted peaks, and the cyan lines are the combined patterns. Kα_2_ peaks are included in the fitting. The Zpex Smile powder contains monoclinic and tetragonal phases of zirconia, indexed with ICDD cards # 86-1450 and 80-0784 respectively. The conventionally sintered sample consists of a tetragonal phase and a small amount of cubic zirconia phase, indexed with ICDD card # 81-1550 [59]. The two-step sintered sample consists mainly of a tetragonal zirconia phase, with a small amount of monoclinic and cubic zirconia phases.

**Figure 14:**
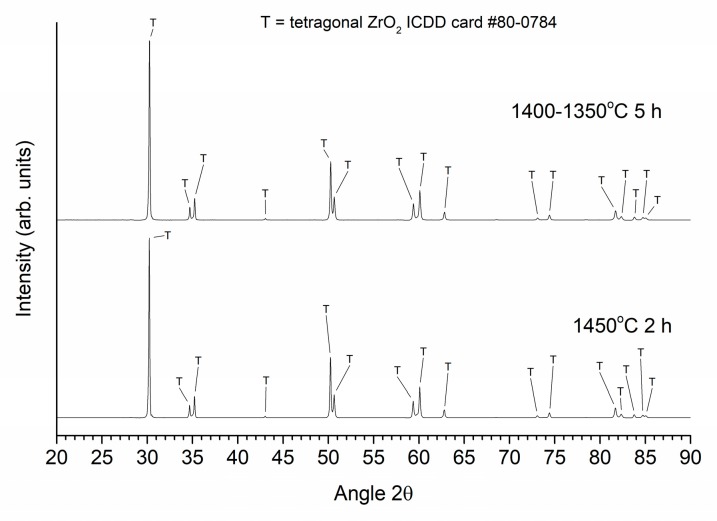
XRD patterns of a conventionally sintered sample and a two-step sintered sample (T1 = 1400 °C, T2 = 1350 °C, t2 = 5 h), both prepared using ball milled powder.

**Figure 15:**
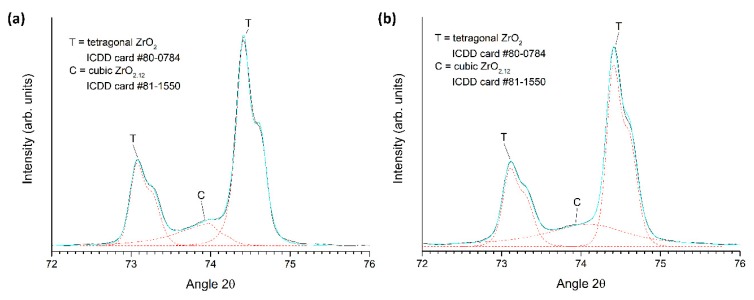
Magnified XRD patterns of (**a**) a conventionally sintered sample and (**b**) a two-step sintered sample (T1 = 1400 °C, T2 = 1350 °C, t2 = 5 h). Both samples were prepared using ball milled powder.

XRD patterns of a sample conventionally sintered at 1450 °C for 2 h and a two-step sintered sample (T1 = 1400 °C; T2 = 1350 °C; t2 = 5 h), both prepared from ball milled powder, are shown in Figure 14. Magnified views of the conventionally sintered and two-step sintered samples in the region 2θ = 72–76° are shown in Figure 15. Peak fitting was carried out as before. Both patterns can be indexed with ICDD card #80-0784 for tetragonal zirconia with a minor cubic zirconia phase present (ICDD card # 81-1550).

Vickers hardness and flexural strength of the conventionally sintered samples (labelled CS) and two-step sintered samples (T1 = 1400 °C, T2 = 1350 °C, t2 = 5–20 h, labelled TSS-5 ~ TSS-20) are given in Table 2. All samples were prepared using as-received powder. The superscript letters indicate significance differences (*p* < 0.05) between the groups i.e. groups with different superscript letters are statistically significantly different from each other. The hardness of the two-step sintered samples increases with sintering time up to 10 h, then decreases. There are significant differences in the hardness values between the conventionally sintered samples and the two-step sintered samples with dwell times of 5 and 20 h. There is no significant change in the flexural strength values with changes in sintering conditions.

**Figure 16:**
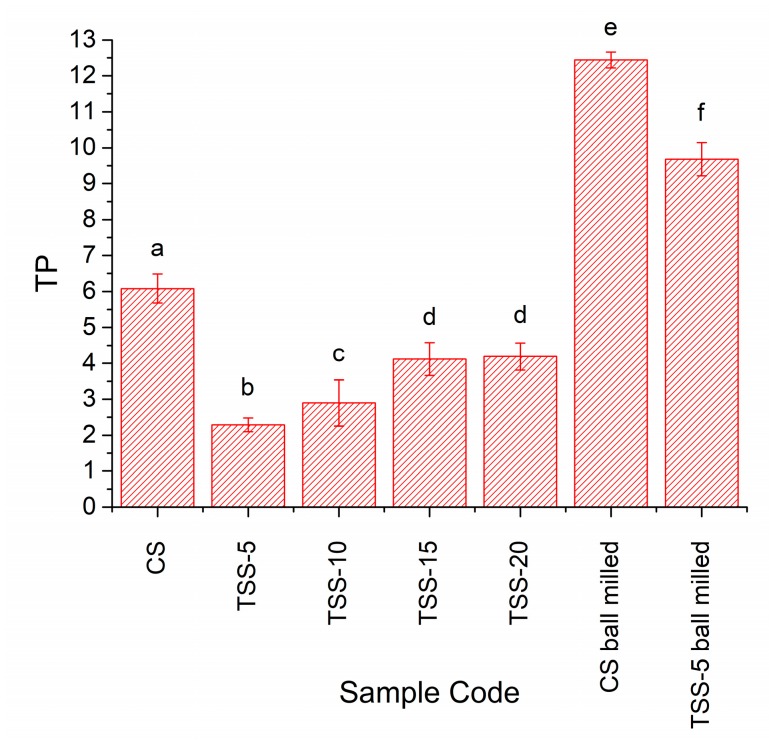
Translucency parameter of conventionally sintered and two-step sintered samples (T1 = 1400 °C, T2 = 1350 °C, t2 = 5–20 h).

Values of translucency parameter TP of the conventionally sintered samples and two-step sintered samples prepared from the as-received powder are shown in Figure 16 (sample codes CS – TSS-20). A higher TP value means higher translucency. The superscript letters indicate significance differences (*p* < 0.05) between the values as before. The conventionally sintered samples have the highest TP value. As the length of the second sintering step increases, the TP value of the two-step sintered samples increases, becoming effectively constant after t2 = 15 h. However, the TP value of the two-step sintered samples does not reach that of the conventionally sintered samples. Values of the translucency parameter, TP, of the conventionally and two-step sintered samples prepared from ball milled powder are also shown in Figure 16. Due to a shortage of samples, one sample was measured five times for each sintering condition. The sample thickness was between 0.5 and 0.6 mm, with the two-step sintered sample being slightly thicker than the conventionally sintered sample (0.57 and 0.54 mm respectively). The conventionally sintered sample has a higher translucency than the two-step sintered sample. The translucency of both these samples is considerably higher than that of the samples prepared from the as-received powder.

**Figure 17:**
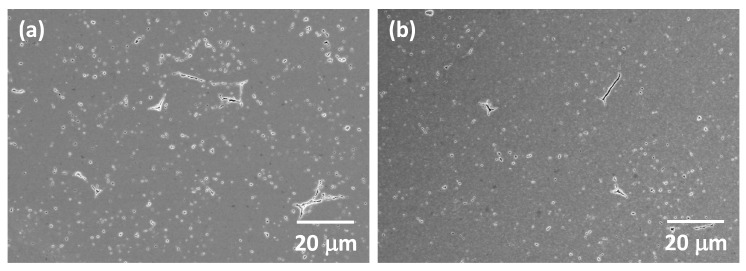
SEM micrographs of (**a**) a two-step sintered sample (T1 = 1400 °C, T2 = 1350 °C, t2 = 20 h) and (**b**) a conventionally sintered sample, both prepared from as-received powder.

Figure 17 shows SEM micrographs of a two-step sintered sample (T1 = 1400 °C, T2 = 1350 °C, t2 = 20 h) and a conventionally sintered sample (both prepared from as-received powder) taken at low magnification. In both cases, large pores can be seen. These are pores formed between the secondary Zpex Smile particles (Figure 1a), which were not eliminated on sintering. The two-step sintered sample contains noticeably more pores than the conventionally sintered samples, despite both samples having the same value of Archimedes density. In particular, more large pores are visible in the two-step sintered sample.

**Figure 18:**
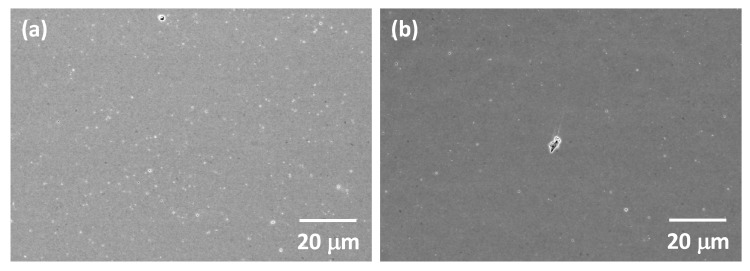
SEM micrographs of (**a**) a two-step sintered sample (T1 = 1400 °C, T2 = 1350 °C, t2 = 5 h) and (**b**) a conventionally sintered sample, both prepared using ball milled powder.

Low magnification SEM micrographs of the two-step sintered and conventionally sintered samples prepared from ball milled powder are shown in Figure 18. The two-step sintered sample is more porous than the conventionally sintered sample. Both samples have noticeably less porosity than their corresponding samples prepared from the as-received powder. In particular, the number of large pores between the secondary particles are reduced. Estimates of area porosity (measured using ImageJ) for the samples in Figure 17 and Figure 18 are given in Table 3. Each value is the mean and standard deviation of measurements from five micrographs. The conventionally sintered samples have less porosity than the two-step sintered samples, and using a ball milled powder causes a noticeable decrease in porosity. The unusually large standard deviation for the conventionally sintered sample using ball milled powder is due to the large pore in Figure 18b.

## 4. Discussion

The two-step sintering technique relies on the difference in kinetics between grain boundary diffusion and grain boundary migration to suppress grain growth in the final stage of sintering [43]. The density of the ceramic after the first step of sintering should be high enough to render the pores unstable against shrinkage [60]. According to the work of Chen and Wang, a density >75% TD should be sufficient [43]. However, in the present work we found that a density >90% TD after the first sintering step was necessary in order to allow sufficient densification during the second sintering step for the samples to attain a value of density equal to that of the conventionally sintered samples (Figure 3 and Figure 6). This density value after the first step is higher than the optimum value of 83% TD (T1 = 1300 °C) found by Mazaheri et al. for Y-TZP [52]. On the other hand, Sutharsini et al. used T1 = 1400 °C (with a corresponding density just below 96% TD) during two-step sintering of tetragonal 3 mol% yttria-stabilized zirconia, similar to the present work [55]. The higher first step sintering temperature used in the present work may be necessary to remove large pores that exist between the secondary particles in the green samples (Figure 17). 

The temperature of the second step, T2, should be high enough to allow sufficient grain boundary diffusion and lattice diffusion of atoms from the grain boundaries to the inter-grain necks (which promotes densification) but not so high as to allow significant grain growth [47]. This is described as the kinetic window by Chen and Wang and also depends on the grain size after the first step [43]. For the samples sintered using Schedule 1 (T1 = 1300 °C and T2 = 1200, 1250 or 1275 °C), sample density increases with T2 and with second step sintering time t2 (Figure 6). However, the density is ≤95% TD, indicating that densification is too slow. For the samples sintered using T1 = 1300 °C and T2 = 1200 °C, grain growth is suppressed during the second stage. However, density levels off after 15 h indicating that densification has been exhausted at this temperature. Increasing T2 allows for continued densification but at the cost of some grain growth (Figure 7). Increasing T1 and T2 (Schedules 2 and 3) allows for improved densification but also induces further grain growth. It is notable that for the samples sintered under Schedule 3 (T1 = 1400 °C, T2 = 1350 °C), densification levels off with increased t2 sintering time, and the maximum density achieved is not greater than that of the conventionally sintered samples. Other workers have also found that it is difficult to sinter to full density using two-step sintering [50].

By comparing Figure 4 and Figure 7, it can be seen that the value of T1 has a larger effect on the grain size than that of T2. It is generally known that the grain growth rate increases with increasing sintering temperature (due to an increase in the grain boundary diffusion coefficient) and with decreasing grain size (due to an increase in the driving force for growth) [61,62]. During the first step of sintering, the sintering temperature is higher than in the second step and the grain size is smaller, due to the fine size of the initial primary particles. Hence, grain growth rates are higher in the first step and are more dependent on the choice of T1.

The possibility of separating the processes of densification and grain growth seems counterintuitive, as both processes depend on grain boundary diffusion and have similar activation energies [63]. There is evidence that triple point junctions may slow down grain growth at lower temperatures, as the mobility of the triple point junctions is lower than that of the grain boundaries [64,65,66,67]. Hence, for temperatures within the kinetic window, grain boundary diffusion is still operative, but grain boundary migration is suppressed, allowing densification without grain growth. Furthermore, the activation energy for sintering may be lower in the final stage of sintering than in the intermediate stage [63,68,69,70]. This will allow densification to continue even at the lower temperature for T2.

The values of flexural strength of both the conventionally sintered and two-step sintered samples (Table 2) are much lower than typical values of flexural strength for zirconia, which are in the range of 600–1000 MPa [5,71,72,73,74]. The manufacturer’s data for the bending strength of this composition is 600 MPa. The large pores in the samples (Figure 17) act as sites for the initiation of cracks, lowering the flexural strength [62,75]. The values of hardness of the conventionally sintered and two-step sintered samples are similar to those in the literature [22,72,76].

According to phase diagrams of the ZrO_2_-Y_2_O_3_ system, the composition of the samples lies in the tetragonal + cubic two phase region at the sintering temperatures used in this study [77,78,79]. Along with the tetragonal phase, a small amount of cubic zirconia was present (Figure 13 and Figure 15), as has also been found in other studies [26,59]. For the two-step sintered samples, holding the sample at T2 = 1350 °C for 5 h does not cause formation of a monoclinic secondary phase, whereas holding the sample at T2 = 1350 °C for 20 h does (Figure 12 and Figure 14). The cubic phase may act as a site for the tetragonal-monoclinic transformation [26]. The formation of a monoclinic secondary phase during the second sintering step should be avoided as it can degrade the mechanical properties [26,80].

As mentioned in the Introduction, light can be scattered at the grain boundaries of a polycrystalline material if it has a non-cubic unit cell. This is due to birefringence—the different crystallographic axes of the crystal have slightly difference values of refractive index due to their different lengths. In a polycrystalline material with randomly oriented grains, each time a ray of light crosses a grain boundary, it will encounter a slight change in the refractive index, and some light will be scattered. This scattering is exacerbated in the case of zirconia due to its relatively high refractive index [12,81]. The amount of scattering can be reduced by making the grain size smaller than the wavelength of the incident light.

For a completely dense birefringent ceramic which does not absorb light, the transmission of light can be estimated using the Rayleigh-Gans-Debye approximation [11,81]:

(5) $$T_{in-line}=\left(1-R\right)exp\left(-\frac{3\pi^{2}\Delta n_{av}^{2}rt}{\lambda^{2}}\right)$$

where *T_in-line_* is the in-line transmission, *R* is the loss due to reflection at the two sample surfaces, ∆*n_av_* is the average birefringence of the material, *λ* is the wavelength of the incident light, *r* is the grain radius and *t* the sample thickness. The in-line transmission decreases as average birefringence, grain radius and sample thickness increase.

An analysis of scattering using the Rayleigh-Gans-Debye approximation [11] showed that the grain size 2*r* of Y-TZP should be ~0.21 μm to achieve an in-line transmission of 2.5% (the average measured value for a range of dental porcelains [17]) for light of wavelength 0.64 μm if ∆*n_av_* is estimated as = 0.03 and *t* = 0.5 mm [81]. The large value of birefringence of Y-TZP may invalidate the Rayleigh-Gans-Debye approximation. Use of the Rayleigh approximation, which is valid only when the size of the grains is lower than the wavelength of the incident light, gave a corresponding grain size of ~0.14 μm [81]. Using the Mie scattering model, which has no restrictions on grain size or birefringence, a grain size of ~0.26 μm was calculated [81]. Whichever model is used, the scattering decreases as grain size decreases. For increasing sample thickness, the grain size needed to achieve the same in-line transmission decreases rapidly [17,81].

Considering the grain size alone, the Y-TZP samples prepared using two-step sintering in the present work should have a higher light transmittance than the conventionally sintered samples. However, from Figure 16 it is clear that this is not the case. The porosity in the samples also has to be taken into consideration. Pores scatter light strongly due to the large difference in refractive index between the material and the gas in the pores [10,14,15,17]. Even 0.1% of porosity could cause a dramatic decrease in light transmittance in alumina [11] and in cubic zirconia [14]. The pore size is also important, with pores <0.1 μm in diameter producing less scattering and pores of diameter comparable to the wavelength of light (0.4–0.7 μm for visible light) producing the most scattering [36]. The translucency parameter, TP, of the two-step sintered samples prepared from as-received powder is initially lower than that of the conventionally sintered samples due to the lower density. As the second step sintering time, t2, increases, the density also increases, leading to an increase in TP due to the reduction in porosity. The conventionally sintered samples and the two-step sintered samples with t2 = 20 h have the same density values (Figure 6). However, SEM micrographs show the two-step sintered sample to have more porosity than the conventionally sintered sample (Figure 17 and Table 3). The difference in porosity may be too small to detect via Archimedes density measurements but can increase scattering and reduce TP. The presence of the secondary monoclinic phase in the two-step sintered samples (Figure 12) will also contribute to scattering as the monoclinic and tetragonal phases have slightly different values of refractive index [81].

In order to further improve the density and translucency of the two-step sintered samples, the number of pores must be further reduced. This can be done by ball milling of the powder [53] or by increasing the cold isostatic pressing pressure in order to break the secondary particles [47]. Both methods lead to an increase in green density of the samples, which is beneficial for achieving high sintered density. Indeed, ball milling of the Zpex Smile powder led to considerable increases in density (Figure 6) and translucency (Figure 16) and to reduced porosity (Figure 18 and Table 3). The reduction in length of the second sintering step also allowed for a further reduction in grain size compared to the conventionally sintered samples (Figure 7). However, due to the combination of slightly increased sample thickness and porosity, the two-step sintered sample still had reduced translucency compared to the conventionally sintered sample (Figure 16). We planned to carry out more two-step sintering experiments using the ball milled powder with longer values of t2 to try and increase sample density further and improve translucency. Unfortunately, persistent maintenance problems with our department’s cold isostatic press meant that we were unable to continue with our experiments. Some samples were uniaxially pressed (i.e., without cold isostatic pressing) and two-step sintered with t2 = 5 and 10 h, but their density was lower (98.4 ± 0.4% and 99.1 ± 0.3% TD respectively) than that of the corresponding cold isostatically pressed two-step sintered samples prepared from as-received powder. Uniaxially pressed samples prepared from ball milled powder could however be conventionally sintered to full density.

## 5. Conclusions

Conventional and two-step sintering experiments were conducted on a commercial Y-TZP powder in order to prepare sintered samples with high density and small grain size. By controlling the sintering parameters (T1 = 1400 °C, t1 = 5 min, T2 = 1350 °C, t2 = 20 h) of the as-received powder, it is possible to prepare two-step sintered samples with equal Archimedes density, reduced grain size and narrower grain size distribution compared to samples conventionally sintered at 1450 °C for 2 h. The conventionally sintered samples consisted of tetragonal zirconia with a cubic zirconia secondary phase, whereas the two-step sintered samples contained an additional monoclinic zirconia secondary phase. The two-step sintered samples have lower translucency than the conventionally sintered samples, due to slight differences in density and possibly to the presence of the monoclinic second phase. By ball milling the as-received powder it is possible to increase sample density to almost theoretical density using two-step sintering whilst still maintaining a small grain size and avoiding the formation of the monoclinic secondary phase. Both conventionally and two-step sintered samples prepared from ball milled powder had increased translucency. However, the two-step sintered sample did not have improved translucency compared to the conventionally sintered sample.

## Figures and Tables

**Table 1:** Two-step sintering schedules of samples prepared from as-received powder.

Schedule	Temperature 1 T1 (°C)	Holding Time 1 t1 (min)	Temperature 2 T2 (°C)	Holding Time 2 t2 (h)
1	1300	5	1200	5–20
			1250	5–20
			1275	5–20
2	1375	5	1325	5–20
3	1400	5	1350	5–20

**Table 2:** Mechanical properties of conventionally sintered and two-step sintered samples prepared from as-received powder.

Sample	Temperature (°C)	Dwell Time (h)	Hardness (GPa)	Flexural Strength (MPa)
CS	1450	2	13.98 ± 0.54 ^a^	381.30 ± 54.66 ^d^
TSS-5	T1-1400; T2-1350	5	13.26 ± 0.47 ^c^	346.38 ± 41.94 ^d^
TSS-10	T1-1400; T2-1350	10	13.84 ± 0.48 ^abc^	345.12 ± 22.70 ^d^
TSS-15	T1-1400; T2-1350	15	13.59 ± 0.89 ^ab^	357.04 ± 49.00 ^d^
TSS-20	T1-1400; T2-1350	20	13.43 ± 0.57 ^c^	402.12 ± 45.36 ^d^

Note: superscript letters a, b, c and d indicate significance differences (p < 0.05) between groups.

**Table 3:** Area porosity of conventionally and two-step sintered samples shown in Figure 17 and Figure 18.

Sample	CS	TSS-20	CS (Ball Milled Powder)	TSS-5 (Ball Milled Powder)
Area porosity (%)	0.111 ± 0.045	0.282 ± 0.099	0.021 ± 0.032	0.032 ± 0.004
